# Association between PM_2.5_ exposure and metabolic syndrome in older population and the mediating effect of amino acids

**DOI:** 10.3389/fpubh.2026.1811045

**Published:** 2026-06-18

**Authors:** Xu Liu, Yunxia Zhou, Yingjie Li, Mu Wang

**Affiliations:** Epidemiology Teaching and Research Office of School of Public Health and Clinical Research Institute of Affiliated Nanhua Hospital, Hengyang Medical School, University of South China, Hengyang, Hunan, China

**Keywords:** amino acids, isoleucine, mediation analysis, metabolic syndrome, PM_2.5_

## Abstract

**Background:**

PM_2.5_ is closely associated with metabolic syndrome (MetS), but the pathways underlying this association remain unclear in older population. This cross-sectional study aimed to investigate the statistical association between short-term PM_2.5_ exposure and MetS in an older population, and to explore whether specific serum amino acids could statistically account for this observed link.

**Methods:**

A total of 1,506 older adults (≥60 years) were recruited from a community in Hengyang City, Hunan Province. The most significant time window of short-term PM_2.5_ exposure (30-day moving average, MA30) associated with MetS was identified using logistic regression. Core serum amino acids associated with both PM_2.5_-MA30 and MetS were screened via elastic net regression. Subsequently, mediation and moderated mediation analyses were employed to assess the indirect statistical association through amino acids and to explore whether this association varied by age or dietary patterns.

**Results:**

The study cohort comprised 396 individuals with metabolic syndrome. A significant positive correlation was observed between PM_2.5_-MA30 exposure and the presence of MetS. Elastic net regression identified isoleucine (Ile) as a co-associated factor. Mediation analysis indicated that the data were consistent with a significant indirect statistical association through Ile (indirect association estimate: 0.007, 95% CI: 0.003, 0.010). No evidence was found that age or dietary patterns significantly moderated this indirect association.

**Conclusion:**

In this cross-sectional study, short-term PM_2.5_ exposure (MA30) was associated with MetS in older adults. This association was statistically accounted for, in large part, by serum isoleucine levels. This association did not appear to vary by age or dietary patterns. These findings generate a novel hypothesis for future longitudinal research on how environmental pollutants may be linked to metabolic dysregulation.

## Introduction

Metabolic syndrome (MetS) comprises a cluster of cardiometabolic risk factors, including abdominal obesity, abnormal blood glucose levels, hypertension, and dyslipidaemia (1). Accompanied by rapid economic development, urbanisation, reduced physical activity and a shift towards high-calorie dietary patterns (2, 3), the prevalence of MetS has risen sharply worldwide and has become a major public health issue. The older population is at a particularly high risk of MetS; declining physiological function, changes in body composition, and the exacerbation of age-related chronic inflammation render them increasingly susceptible to metabolic disorders. Numerous studies have demonstrated that the prevalence of MetS increases significantly with age (4, 5). As a country experiencing rapid population ageing, China faces particularly severe metabolic health challenges among its older population. Data from 2015 indicated that the prevalence of MetS among older adults in China had reached as high as 36.9% (6), significantly higher than the median prevalence among the global older population (21%) (7), and this proportion continues to rise (8). Therefore, an in-depth investigation into the modifiable risk factors statically associated with the onset and progression of MetS in older adults is crucial for formulating targeted primary prevention strategies in public health.

Among the many environmental risk factors, fine particulate matter (PM_2.5_) poses a significant health threat due to its widespread exposure and toxicological effects (9–11). Substantial epidemiological evidence consistently indicates that PM_2.5_ exposure is correlated with an increased risk of MetS and its individual components (12–15). However, evidence regarding this association remains inconsistent, with conflicting results reported in recent reviews (16, 17). Whilst early studies focused primarily on developed countries (18, 19), significant progress has been made in recent research on older population in China; for example, studies have revealed the effects of ozone on the lipid profile (20) and identified the mediating role of apolipoprotein B (ApoB) in the association between PM_2.5_ components and metabolic syndrome (21). Although these findings provide valuable insights into lipid-related mechanisms, the broader biological pathways linking PM_2.5_ exposure to the exacerbation of metabolic dysregulation remain incompletely elucidated. To systematically elucidate the potential pathways by which PM_2.5_ affects metabolic health, it is particularly important to identify and validate other key biomarkers. As one of the key biochemical pathways in metabolic syndrome, amino acid metabolism has attracted widespread attention (22).

Amino acids are not only the building blocks of protein synthesis, but also important signalling molecules and substrates for energy metabolism. Imbalances in their homeostasis are closely associated with various metabolic disorders. Large-scale prospective cohort studies have found that elevated levels of circulating branched-chain amino acids (leucine, valine, and isoleucine) and aromatic amino acids (tyrosine, phenylalanine, and tryptophan) serve as biomarkers for predicting related metabolic diseases (23–25). Cross-sectional studies have also observed that serum levels of various amino acids are significantly higher in patients with MetS than in healthy individuals (26). Notably, levels of certain amino acids (such as serine, asparagine, glutamine and glycine) appear to be negatively correlated with components of MetS (23, 27, 28). Furthermore intervention studies suggest that arginine may improve metabolic parameters such as insulin sensitivity (29, 30). Taken together, this evidence demonstrates a complex and multifaceted relationship between amino acid metabolism and MetS. At the same time, although the relevant literature is limited, epidemiological studies have suggested that exposure to PM_2.5_ may lead to disturbances in amino acid metabolism (31–34). However, existing research findings are inconsistent; for example, different studies have reported conflicting results regarding the direction of the association between specific amino acid levels and PM_2.5_ exposure (32, 35).

The heterogeneity observed in the aforementioned associations suggests that the pathway through which PM_2.5_ exposure influences MetS risk via amino acid metabolism may be modulated by significant individual factors. Identifying these modifiers is crucial for elucidating mechanistic heterogeneity and identifying high-risk subgroups. Firstly, age is considered a key moderator of the health effects of environmental exposures. Biological ageing is accompanied by a decline in antioxidant capacity (reduced glutathione synthesis and decreased activity of superoxide dismutase and catalase), leading to redox imbalance (36). This imbalance may not only exacerbate the metabolic demands and depletion of amino acids within antioxidant pathways (37), but also progressively worsen a state of chronic low-grade inflammation, thereby promoting the onset of metabolic diseases (38). Therefore, theoretically, differences in physiological status within the same age group of older adults, resulting from variations in the ageing process, may significantly modulate their metabolic susceptibility to PM_2.5_ exposure and the associated amino acid metabolic responses. Secondly, dietary patterns represent another key modifiable factor. Diet directly supplies the body with macronutrients and micronutrients, forming the foundation of antioxidant, anti-inflammatory and metabolic defence systems (39). For example, observational studies have found that higher levels of vitamin E in the body are associated with a reduced adverse impact of PM_2.5_ exposure on blood pressure elevation and vascular endothelial function (40). Intervention studies have also shown that supplementation with antioxidant nutrients can, to some extent, mitigate the trend towards elevated biomarkers of inflammation and oxidative stress induced by air pollution (41). Conversely, dietary patterns characterized by high levels of saturated fat, highly refined carbohydrates and highly processed foods may impair the body’s defences, producing an adverse synergistic effect with PM_2.5_ exposure (42). Of particular relevance is the fact that, as essential amino acids, branched-chain amino acids (BCAAs) must be obtained from the diet, and animal studies suggest that a BCAA-rich diet may contribute to the development of metabolic diseases by upregulating the expression of specific pathways (43, 44). Consequently, dietary patterns may mitigate or exacerbate the metabolic health effects of PM_2.5_ exposure, both by influencing amino acid intake and metabolism, and by modulating systemic oxidative stress and inflammatory states.

In summary, although there is evidence linking “PM_2.5_ and MetS,” “amino acids and MetS,” and “PM_2.5_ and amino acids,” and although age and dietary patterns may moderate this relationship, to date there has been a lack of research that places all three within the same epidemiological framework to systematically examine “the mediating role of serum amino acids in the PM_2.5_-MetS association” whilst simultaneously investigating “the moderating effects of age and dietary patterns on this mediating pathway.” Consequently, this study proposes the following exploratory hypotheses for a community-based older population in China: 1) there is a statistical association between PM_2.5_ exposure and MetS status. 2) This observed association may be statistically accounted for, in part, by alterations in the serum amino acid profile. 3) This potential statistical mediation pattern might vary according to an individual’s age and dietary patterns. To explore these hypotheses, this study aimed: firstly, to examine the statistical association between PM_2.5_ exposure across different time windows and MetS status. Secondly, to identify candidate serum amino acids concurrently associated with both PM_2.5_ exposure and MetS. Thirdly, to test the specific hypothesis that the identified amino acids statistically mediate the PM_2.5_-MetS link, and to quantify the proportion of the total association accounted for by this potential mediation. Fourthly, to explore whether any such statistical mediation pattern appears to differ by age or dietary patterns using moderated mediation analysis.

## Methods and measurements

### Study population

This was a cross-sectional study. Between June and October 2022, we recruited participants through public notices posted in the community, a total of 1,655 residents aged 60 years and over, who had lived in a community in Hengyang City, Hunan Province, for at least 2 years, volunteered to take part in the project. The age threshold of 60 years was adopted based on a consensus in the epidemiological literature on MetS in China, which identifies this age group as having a high prevalence of the condition (45, 46). At baseline, we collected data through structured questionnaires and face-to-face interviews (covering sociodemographic characteristics, behaviours, and lifestyle factors), and performed physical measurements and biological sample collection. All procedures were conducted by trained staff and all participants provided written informed consent. After excluding participants with missing demographic data or test indicator data, a total of 1,506 participants were included in the final analysis (Supplementary Figure S1). According to China’s chronic disease surveillance data, the prevalence of MetS among the older population stands at 36.900% (35). Based on the formula for calculating the minimum sample size required for cross-sectional studies (Equation 1), the minimum sample size required for this study is 994 individuals. Considering an 80% response rate, the minimum sample size required is 1,243 individuals. The final sample size enrolled in this study was 1,506 participants, meeting the minimum sample size requirement for a cross-sectional study. This study was approved by the Ethics Committee of Affiliated Nanhua Hospital, University of South China (2022-KY-167). All participants provided written informed consent, and the study was conducted in accordance with the principles of the Declaration of Helsinki.


$$n=({Z_{\frac{\alpha }{2}}}^{2}\times \pi \times (1-\pi ))/\delta^{2}$$ (1)

In Formula 1, a 95% confidence level is selected, where “n” denotes the sample size required for the cross-sectional study; the corresponding “ $Z_{\alpha /2}$ ” value is 1.96; “*π*” represents the prevalence of MetS within the older population; “*δ*” denotes the permissible error, set at 3% in this study.

### Clinical measurements

Clinical baseline data were collected by trained staff in community hospitals through face-to-face interviews using a structured questionnaire. The questionnaire covered general demographic characteristics (gender, age, educational attainment, marital status), lifestyle factors (smoking, alcohol consumption, regular physical exercise) and dietary frequency surveys (including weekly intake of fresh vegetables, fruit, meat/eggs/dairy products, soy products, garlic, chilli and fried foods). The relevant variables were defined in detail in the structured questionnaire: Smoking was defined as consuming one or more cigarettes per day for six consecutive or cumulative months or more. Drinking was defined as drinking more than 50 mL per day for more than 1 year. Regular physical exercise refers to engaging in physical activity on average three or more times per week, with each session lasting over 30 min. Dietary frequency survey refers to respondents’ weekly consumption frequency of the following: Fresh vegetables (approximately 100 g per serving); Fresh fruit (approximately 150-200 g per serving); Meat/eggs/dairy (equivalent to 50 g meat, 1 egg or 300 mL milk per serving); Soy products (equivalent to 100 g tofu or 300 mL soya milk per serving); garlic (approximately 3–5 cloves per serving); chilli peppers (equivalent to 1 fresh chilli or 3–5 dried chillies per serving); fried foods (equivalent to 1 fried dough stick or 150 g of fried chicken pieces per serving).

The physical examination included measurements of height, weight, waist circumference and blood pressure. All procedures were carried out by trained personnel in accordance with the “Methods for Anthropometric Measurements in Health Monitoring” (WS/T 424–2013) of the “Health Industry Standards of the People’s Republic of China.” To ensure the accuracy of biochemical tests, participants fasted for at least 8 h prior to blood collection. Blood samples were drawn from the antecubital vein at the community hospital between 7:00 and 9:00 the following morning. Blood samples were collected in vacuum tubes and centrifuged at 3000 g for 10 min at 25 °C to separate the serum. The parameters tested included fasting plasma glucose (FPG), total cholesterol (TC), triglycerides (TG), serum creatinine (Scr), high-density lipoprotein cholesterol (HDL-C), low-density lipoprotein cholesterol (LDL-C), alanine aminotransferase (ALT) and aspartate aminotransferase (AST).

### Exposure measurement of PM_2.5_

In this study, ambient PM_2.5_ exposure was estimated using data from the Hengyang Meteorological Monitoring Station, situated approximately 2 km from the participants’ residential community. This approach, which employs fixed-site monitoring data as a proxy for community-level exposure, is commonly adopted in large-scale epidemiological studies where personal exposure monitoring remains impractical (21, 47). It employs fixed-site monitoring data as a practical proxy for community-level exposure when personal exposure monitoring remains impractical. The primary objective of this cross-sectional framework is to explore potential statistical mediating pathways rather than to precisely quantify absolute individual exposure doses. Consequently, data derived from fixed monitoring points serve as a reliable surrogate indicator for comparing relative recent exposure levels among participants. To estimate these relative exposure levels, we extracted daily average PM_2.5_ concentration data covering the day of each participant’s medical examination alongside specific periods prior to that date. We then calculated the moving average concentrations of PM_2.5_ exposure for the 7, 14, 30, 60, 90 and 120 days preceding each participant’s medical examination, and labelled these as PM_2.5_-MA7, PM_2.5_-MA14, PM_2.5_-MA30, PM_2.5_-MA60, PM_2.5_-MA90 and PM_2.5_-MA120, and used these as indicators of individual PM_2.5_ exposure levels. Metabolic syndrome is inherently a chronic condition that typically requires several years or even decades to fully manifest. This study, drawing on relevant epidemiological literature (47–49), utilises short-term (7–120 days) moving average exposure measures, it does not capture the full duration of PM_2.5_ exposure over the entire period of MetS development. Within this framework, we hypothesize that, during the maintenance or progression of MetS, recent exposure to PM_2.5_ (7–120 days) may act as an additional environmental stressor, exacerbating systemic oxidative stress, inflammatory responses and metabolic dysregulation, thereby accelerating pre-existing metabolic dysregulation at an early clinical stage. Consequently, the PM_2.5_-MetS association identified in this study should be cautiously interpreted as a cross-sectional statistical association between recent exposure and the current disease status. This association may reflect the role of recent exposure during the disease progression phase, whereas exposure occurring earlier in the disease course or over a longer period was not captured by the measurement window of this study. The selection of a 7-120-day exposure window for PM_2.5_ was not intended to establish the aetiological role of long-term PM_2.5_ exposure, but rather to verify whether a statistical association exists between recent PM_2.5_ exposure (7–120 days) and the current status of MetS.

### Definition of MetS

Based on participants’ waist circumference, blood pressure, fasting blood glucose (FBG), triglycerides (TG) and high-density lipoprotein cholesterol (HDL-C) as recorded during clinical physical examinations, or on information regarding the presence of hypertension, type 2 diabetes or dyslipidaemia, the prevalence of MetS among study participants was determined in accordance with the International Diabetes Federation’s definition of MetS in Asian populations: (i) central obesity (waist circumference (WC) ≥80cm in females and ≥90cm in males); (ii) high BP (systolic BP ≥130mmHg , diastolic BP ≥85mmHg , or treatment for previously diagnosed with hypertension); (iii) elevated FBG (levels of FBG ≥100mg/dL or previously diagnosed with type 2 diabetes); (iv) high TG (levels of TG ≥150mg/dL or specific treatment for lipid abnormalities); (v) Low HDL-C (levels of HDL-C <50mg/dL in females and <40mg/dL in males, or specific treatment for lipid abnormalities). Individual with three or more of the above components was diagnosed as MetS case (50).

### Mediation and moderated mediation analyses

The mediation model integrates independent variables, dependent variables and mediating variables within a single statistical framework, enabling a quantitative exploration of the extent to which an observed association between an exposure and an outcome might be statistically accounted for by a proposed mediator. It decomposes the total observed association into direct and indirect associations mediated by the mediating variable, thereby going beyond traditional analyses that merely describe the association between the two variables and can offer statistical evidence that is consistent with hypothesised biological pathways (51, 52). This characteristic has led to the widespread use of mediation analysis models in metabolomics and epidemiological research (53, 54). In this study, the simple mediation model was conducted using Model 4 in SPSS Process (Supplementary Figure S2), whereby the total association of the independent variable (X) on the dependent variable (Y) was decomposed into direct and indirect associations. The direct association refers to the influence that X exerts on Y when the mediating variable (M) is held at a specific level. The indirect association, also interpreted as the potential mediating pathway, refers to the component of the X-Y association that operates through M. The 95% confidence interval (95% CI) for the indirect association is estimated; if this confidence interval does not include 0, it is inferred that the data are consistent with a significant statistical mediation by M between X and Y (55). To examine whether the proposed indirect statistical pathway varied with the moderator variable (W), this study constructed a moderated mediation model using Model 59 in SPSS Process (Supplementary Figure S3). This model explores whether the strength of the indirect association varies across different levels of W. The results primarily consist of conditional direct associations, conditional indirect associations, and between-group comparisons of conditional indirect associations. The between-group comparison of the conditional indirect association involves comparing the indirect association values at different levels of W to test for statistical differences between them. If the 95% CI for this difference does not include 0, it indicates that the strength of the indirect association changes significantly with variations in W, which would suggest the presence of a moderating effect (56). The mediation and moderated mediation analyses applied in this paper are based on cross-sectional data using short-term exposure measures. Within this framework, the estimated “indirect association” can only reveal a statistical pattern of co-occurrence (i.e., X is associated with M, M is associated with Y, and part of the X-Y association is statistically mediated by M). It cannot prove that M is the causal mechanism or biological pathway through which X leads to Y. Therefore, the results of these analyses should be understood as identifying statistically significant and biologically plausible potential mediation patterns, which may provide specific hypotheses for future longitudinal or experimental studies aimed at causal inference. In this study, amino acids will be included as the mediating variable in the mediating effect model, whilst participants’ age and different dietary patterns will be included as moderating variables in the moderated mediating effect model.

### Screening for mediating variables

Amino acid metabolism has attracted widespread attention as a key biological mechanism linking environmental stimuli and metabolic homeostasis (22). Therefore, this study hypothesizes that amino acid metabolism may act as a key mediating variable in the relationship between PM_2.5_ exposure and the risk of metabolic syndrome. In this study, liquid chromatography–tandem mass spectrometry (LC–MS/MS) was employed in multiple reaction monitoring (MRM) mode for the analysis of serum amino acids. A total of 34 amino acids were identified (His, Hyp, 3MHis, 1MHis, PEtN, Asn, Arg, Tau, Ans, Ser, Gln, Gly, EtN, Asp, Cit, Sar, Glu, bAla, Thr, Ala, Hcit, Aad, bAib, Pro, Abu, Cys, Tyr, Met, Val, Ile, Leu, Phe, Trp, Lys). However, given the high multicollinearity among amino acids (Supplementary Figure S4), we selected an elastic net regression model to identify amino acids that may might statistically mediate the correlation between “PM_2.5_” and “MetS,” thereby avoiding potential analytical bias. The elastic net regression model is a hybrid regularised linear regression algorithm based on the least squares method. By combining both L1 regularisation (Lasso) and L2 regularisation (Ridge regression), it possesses the feature selection capability of Lasso alongside the resistance to multicollinearity of Ridge regression, thereby yielding more robust estimates. The amino acids ultimately selected as candidate mediating variables were determined based on the *λ*.1se criterion, which represents the optimal balance between model complexity and predictive accuracy, thereby preventing overfitting (57).

### Covariates

This study aims to explore the potential mechanisms linking PM_2.5_ exposure to the risk of MetS. Based on a review of the relevant literature (58–61), it has been found that PM_2.5_ exposure may affect the risk of developing MetS via two main pathways: (1) Traditional metabolic pathways, whereby PM_2.5_ exposure correlates with systemic inflammation and oxidative stress. This state further correlates with a series of metabolic disorders, such as dyslipidaemia (TC, LDL-C), liver stress (ALT and AST), and obesity (BMI). These dysregulations are themselves core components of MetS or strongly associated phenotypes. (2) Other pathways, represented by amino acid metabolism: PM_2.5_ may increase the risk of MetS by affecting other biological systems (such as the homeostasis of amino acid metabolism); this pathway may be partially independent of changes in traditional metabolic pathways, as illustrated in Supplementary Figure S5. Based on this framework, the present study aimed to preliminarily investigate whether PM_2.5_ demonstrates an independent statistical association with MetS risk alongside variations in amino acid metabolism. Consequently, we actively adjusted for key indicators representing traditional metabolic pathways (BMI, TC, LDL-C, ALT, and AST) in our models. Consequently, the effect sizes reported in this study regarding the association between PM_2.5_ exposure and MetS should be cautiously interpreted as residual potential associations remaining after statistically controlling for specific indicators of traditional metabolic pathways, rather than the total effect of PM_2.5_ on MetS. Specifically, the covariates requiring adjustment in this study should include gender, age, educational attainment, marital status, smoking status, drinking status, regular physical exercise, BMI, renal function status, LDL-C, serum ALT and AST, TC, and weekly frequency of consumption for fresh vegetables, fresh fruit, meat/eggs/dairy products, soy products, garlic, chilli peppers, and fried foods. Renal function was assessed using estimated glomerular filtration rate (eGFR), which is considered the best indicator for evaluating renal function (62). eGFR is calculated using the modified renal diet study formula (63): eGFR:175×Scr (mg/dL)^−1.234^ × age^−0.179^ (male), 175 × Scr (mg/dL)^−1.234^ × age^−0.179^ × 0.79 (fmale). Based on relevant studies (64), eGFR was categorised into three groups: normal kidney function: eGFR $\geq 90\;\mathrm{mL}/\text{minper}\;1.73\;m^{2}$ , mildly impaired kidney function: eGFR between $60-90\;\mathrm{mL}/\text{minper}\;1.73\;m^{2}$ , and significantly impaired kidney function: eGFR $<60\;\mathrm{mL}/\text{minper}\;1.73\;m^{2}$ .

### Statistical analysis

Descriptive analysis was conducted using SPSS 27.0 statistical software to examine the general demographic characteristics, biochemical indicators, dietary intake frequency, and contaminant concentrations within the study population. Quantitative data are presented as $\bar{x}\pm s$ , while categorical data are expressed as frequency (proportion).

The main analysis was conducted in three steps. Step1: To identify the PM_2.5_ exposure window most closely associated with MetS, we constructed a series of multivariable logistic regression models in this study. In each model, MetS was the dependent variable, whilst PM_2.5_ concentrations across different exposure windows (MA7, MA14, MA30, MA60, MA90, and MA120) served as independent variables to assess their association with MetS. All models were uniformly adjusted for the same confounding factors, including gender, age, educational attainment, marital status, smoking status, drinking status, regular physical exercise, BMI, renal function status, LDL-C, serum ALT and AST, TC, and weekly frequency of consumption of fresh vegetables, fresh fruit, meat/eggs/dairy products, soy products, garlic, chilli peppers, and fried foods. The PM_2.5_ exposure window showing a statistically significant association with MetS was selected as the exposure measure for all subsequent analyses. To ensure the statistical robustness of the final model, we assessed multicollinearity between the selected PM_2.5_ exposure window and all included covariates using the variance inflation factor (VIF). The VIF values for all variables were below 5, indicating the absence of severe multicollinearity. Furthermore, to investigate whether the effects observed within this key time window were independent of other co-occurring air pollutants, we subsequently constructed dual-pollutant models, incorporating NO_2_, SO_2_ and O_3_, respectively, alongside the selected PM_2.5_ exposure time window into the models. Step2: To identify key amino acids associated with both PM_2.5_-MA30 exposure and MetS status, this study employed elastic net regression. This model is effective in handling high-dimensional and multicollinear data, and incorporates variable selection capabilities. Specifically, the analysis was conducted using the “glmnet” package in R, with models fitted using PM_2.5_-MA30 (continuous variable) and MetS status (binary variable) as outcomes, respectively. All amino acid data were log-transformed and included alongside the same covariates as in the first step. Key model settings included: fixing the mixing parameter *α* at 0.5 to balance the penalties of Lasso and Ridge regression. The regularisation path was determined via 10-fold cross-validation. The optimal *λ* value (*λ* = 1.1se) was subsequently selected based on the “one standard error rule” to obtain a more parsimonious and robust model. Furthermore, we set differential penalty factors, applying penalties only to amino acid variables (factor set to 1), whilst setting the penalty factor for all covariates to 0 to ensure that the latter’s contributions were not excluded. Finally, we identified the amino acids with non-zero coefficients at “*λ*.1se” across both models by taking their intersection, thereby determining the candidate variables for subsequent mediation analysis. Step3: The amino acids identified in Step2 were included as mediating variables in a simple mediation model (Model 4), whilst adjusting for the same covariates as in Step1. To test the robustness of the mediation pathway and to explore potential moderating effects of age and dietary patterns, we further conducted a mediation analysis with moderation (Model 59). Different dietary patterns were extracted from the weekly consumption frequencies of different food groups (vegetables, fruit, meat/eggs/dairy, soy products, garlic, chilli, fried foods) via principal component analysis with varimax rotation. Individual factor scores for these patterns, along with age, were then tested as moderators. For all mediation and moderated mediation models, effect estimates were considered statistically significant if their 95%CI (based on 5,000 bootstrap repetitions) did not include 0. In models with age as a moderator, we controlled for all covariates identical to those in the first-step model, excluding age. Meanwhile, in models with dietary patterns as moderators, we controlled for all covariates except for all dietary frequencies (weekly frequency of consumption for fresh vegetables, fresh fruit, meat/eggs/dairy, soy products, garlic, chilli peppers, and fried foods).

## Result

### Characteristic of samples

Table 1 showed the demographic characteristics of the participants. A total of 1,506 older adults were included, with an average age of 72.155 years. Women accounted for 57.171, and 70.053% had an educational level of primary school and below. Most participants did not drink (91.102%), while 5.312% of the population have the habit of smoking. Most participants (82.138%) were married, and 77.556% had normal kidney function. 82.736% of the population regularly engage in physical exercise. Regarding the frequency of dietary intake over the preceding week among the study cohort, fresh vegetables were consumed most frequently, averaging 8.677 times per week. Meat/eggs/dairy products followed (7.570 times), while fried foods were consumed least frequently (0.628 times).

**Table 1:** General characteristics of the study population (*N* = 1,506).

Characteristics	Classification	$\bar{x}\pm s$	*n* (%)
PM_2.5_lag7		16.566 ± 7.764	—
PM_2.5_lag14		16.408 ± 8.093	—
PM_2.5_lag30		20.372 ± 11.798	—
PM_2.5_lag60		25.080 ± 14.817	—
PM_2.5_lag90		28.138 ± 13.806	—
PM_2.5_lag120		33.752 ± 6.060	—
age(years)		72.155 ± 7.113	—
BMI		23.407 ± 3.187	—
ALT (U/L)		21.736 ± 15.456	—
AST (U/L)		22.687 ± 10.299	—
LDL-C (mmoL/L)		3.450 ± 35.862	—
TC (mmol/L)		4.579 ± 0.960	—
Fresh vegetables		8.677 ± 4.383	
Fresh fruit		4.431 ± 2.745	
Meat, eggs and dairy		7.570 ± 4.452	
Soy products		2.759 ± 1.899	
Fried foods		0.628 ± 3.930	
Garlic		6.461 ± 4.168	
Chilli peppers		5.728 ± 4.089	
MetS	Yes	—	396 (26.295%)
	No	—	1,110 (73.705%)
Gender	Male	—	645 (42.829%)
	Female	—	861 (57.171%)
Renal function status	Normal	—	1,168 (77.556%)
	Mild decline	—	260 (17.264%)
	Significant decline	—	78 (5.179%)
Regular exercise	Yes	—	1,246 (82.736%)
	No	—	260 (17.264%)
Drinking status		—	
	Yes	—	134 (8.898%)
	No	—	1,372 (91.102%)
Smoking status	Smoking all the time	—	80 (5.312%)
	Have quit smoking	—	221 (14.675%)
	Never smoked	—	1,205 (80.013%)
Status of education	Primary school and below	—	329 (21.846%)
	Junior high school	—	726 (48.207%)
	High school/vocational High Schools	—	351 (23.307%)
	College or above	—	88 (5.843%)
	Unknown	—	12 (0.797%)
Marital status	Unmarried	—	13 (0.863%)
	Married	—	1,237 (82.138%)
	Divorced/widowed	—	256 (16.999%)

SD, standard deviation; ALT, alanine aminotransferase; AST, aspartate aminotransferase; LDL-C, low-density lipoprotein cholesterol; TC, total cholesterol; PM_2.5_, fine particulate matter; PM_2.5_(lag7), PM_2.5_ lagged by 7 days; PM_2.5_(lag14), PM_2.5_ lagged by 14 days PM_2.5_(lag30), PM_2.5_ lagged by 30 days; PM_2.5_(lag60), PM_2.5_ lagged by 60 days; PM_2.5_(lag90), PM_2.5_ lagged by 90 days; PM_2.5_(lag120), PM_2.5_ lagged by 120 days; MetS, metabolic syndrome.

This study included 396 participants with MetS and 1,110 without MetS (Table 1). The mean values for PM_2.5_-MA7, PM_2.5_-MA14, PM_2.5_-MA30, PM_2.5_-MA60, PM_2.5_-MA90, and PM_2.5_-MA120 were 16.566 μg/m^3^, 16.408 μg/m^3^, 20.372 μg/m^3^, 25.080 μg/m^3^, 28.138 μg/m^3^, 33.752 μg/m^3^ respectively. Supplementary Table S1 shows the mean serum amino acid concentrations (His, Hyp, 3MHis, 1MHis, PEtN, Asn, Arg, Tau, Ans, Ser, Gln, Gly, EtN, Asp, Cit, Sar, Glu, bAla, THr, Ala, Hcit, Aad, bAib, Pro, Abu, Cys, Tyr, Met, Val, Ile, Leu, Phe, Trp, Lys) in the study population.

### Associations of PM_2.5_ with MetS

To identify the most influential time window for PM_2.5_ exposure associated with metabolic syndrome, this study assessed the association between exposure to PM_2.5_ over different time windows (MA7, MA14, MA30, MA60, MA90, MA120, and MetS).

**Figure 1:**
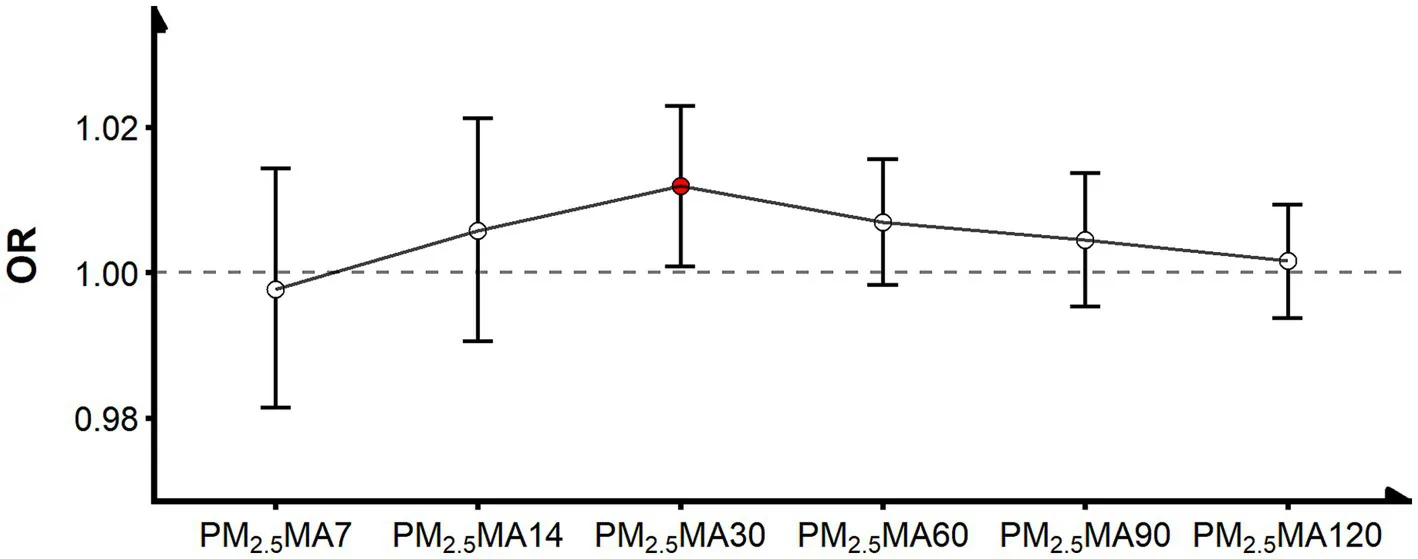
Trend variations in the association between PM_2.5_ exposure and metabolic syndrome risk across different time windows.

Logistic regression models were constructed after adjusting for participants’ baseline data, health behaviour factors, dietary frequency and key indicators representing traditional metabolic pathways. The analysis revealed that the strength of the association between PM_2.5_ exposure and metabolic syndrome varied across different exposure time windows. There is a weak but statistically significant positive residual association between PM_2.5_ (MA30) exposure and the ambient status of developing metabolic syndrome (OR = 1.012, 95% CI: 1.001–1.023, *p* = 0.033). This value reflects the conditional association after controlling for the set of covariates. The VIF values for all variables are < 5 (Supplementary Table S2). Furthermore, in the dual-pollutant model, the statistical association between PM_2.5_ (MA30) and metabolic syndrome remained relatively stable even after further adjusting for NO_2_, SO_2_ and O_3_ separately (Supplementary Table S3), suggesting that PM_2.5_ plays an independent role to some extent. Given the nature of cross-sectional study designs and short-term exposure measurements, this association must be interpreted with extreme caution. It merely indicates a statistically significant correlation between recent PM_2.5_ exposure levels and current MetS status in older adults. This correlation may be driven by a variety of complex, unmeasured temporal relationships. Our decision to select PM_2.5_ (MA30) for subsequent mediation analysis was based on the statistical correlation demonstrated by this time window during the analysis process, with the aim of further exploring whether amino acids play a potential mechanistic role in this cross-sectional association. Figure 1 and Table 2 illustrate the trends in the odds ratios (OR) for MetS and their 95%CI across different PM_2.5_ exposure windows. As shown, the association peaked for the 30-day moving average of PM_2.5_ and then declined gradually as the moving average window extended further into the past. No significant association was observed for shorter or longer exposure time windows; although PM_2.5_-MA30 was statistically significant for MetS, the effect size was small. This may reflect the time-biological window through which PM_2.5_ influences metabolic processes, or it may suggest that a larger sample size is required to obtain robust estimates.

**Table 2:** Association analysis of PM_2.5_ exposure at different latency periods with the current status of MetS Risk.

PM_2.5_ exposure at different latency periods	*β*	SE	OR Value	95% CI	*p*
lag7	−0.002	0.008	0.998	0.981–1.014	0.783
lag14	0.006	0.008	1.006	0.991–1.021	0.456
lag30	0.012	0.006	1.012	1.001–1.023	0.033
lag60	0.007	0.004	1.007	0.998–1.016	0.114
lag90	0.004	0.005	1.004	0.995–1.014	0.338
lag120	0.002	0.004	1.002	0.994–1.009	0.693

All binary logistic regression models were adjusted for general demographic characteristics: gender, age, educational attainment, marital status; lifestyle factors: smoking status, drinking status, and regular physical exercise; health behaviour factors (BMI, kidney function status, LDL-C, serum ALT, serum AST, TC); Dietary frequency survey (weekly intake frequency of fresh vegetables, fresh fruit, meat, eggs and dairy products, soy products, garlic, chilli peppers, and fried foods).

### Amino acid screening based on elastic net regression analysis

**Figure 2:**
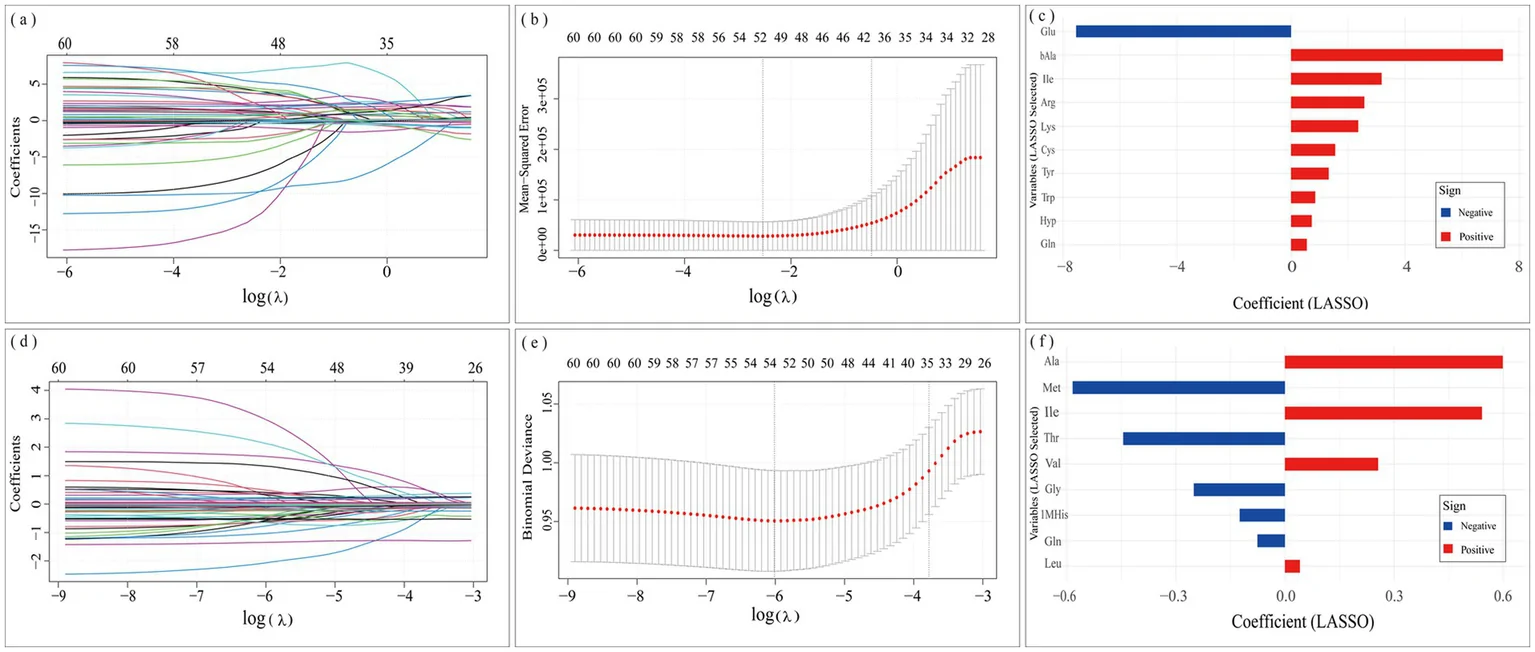
Amino acid screening process based on elastic net regression. Elastic net regression was employed to analyse the associations between amino acids and both PM_2.5_ exposure and metabolic syndrome (MetS). **(a)** Coefficient path diagram of the PM_2.5_ model; **(b)** Cross-validation diagram of the PM_2.5_ model; **(c)** Feature selection diagram for amino acids screened by the PM_2.5_ model; **(d)** Coefficient path diagram of the MetS model; **(e)** Cross-validation diagram of the MetS model; **(f)** Feature selection diagram of amino acids screened by the MetS model. All elastic net regression analyses adjusted for general demographic characteristics: gender, age, educational attainment, marital status; lifestyle factors: smoking status, drinking status, and regular physical exercise; health behaviour factors (BMI, kidney function status, LDL-C, serum ALT, serum AST, TC); Dietary frequency survey (weekly intake frequency of fresh vegetables, fresh fruit, meat/eggs/dairy products, soy products, garlic, chilli peppers, and fried foods).

Establish an elastic net regression model (Figure 2) to screen amino acids associated with PM_2.5_ exposure and MetS, controlling for general demographic characteristics, health behaviour factors, physical examinations, and dietary frequency, using *λ*.1se (maximum regularisation parameter within one standard error). For the PM_2.5_ model (Supplementary Table S4), ten associated amino acids were identified at λ.1se = 0.618. These included Hyp (coefficient = 0.726), bAla (coefficient = 7.433), Arg (coefficient = 2.571), Gln (coefficient = 0.553), Cys (coefficient = 1.548), Tyr (coefficient = 1.322), Ile (coefficient = 3.175), Trp (coefficient = 0.847), and Lys (coefficient = 2.355), all of which exhibited significant positive statistical correlations. For the MetS model (Supplementary Table S5), 9 relevant amino acids were screened at λ.1se = 0.023. Among these, Ala (coefficient = 0.599), Val (coefficient = 0.256), Ile (coefficient = 0.542), and Leu (coefficient = 0.041) exhibited significant positive correlations. Two amino acids were jointly identified by both models: Gln and Ile, which were therefore selected as candidate variables for subsequent mediation analysis.

### Mediating role of amino acids in “PM_2.5_(MA30)-MetS” associations

In Table 3 and Supplementary Table S6, PM_2.5_-MA30 showed a significant positive correlation with serum Ile levels (*β* = 0.005, 95% CI: 0.004, 0.006), whilst elevated Ile levels were significantly associated with MetS status in the current study population (*β* = 1.216, 95% CI: 0.636, 1.796). The indirect association via Ile was 0.007 (95% CI: 0.003, 0.010), accounting for 57.895% of the total observed association. Upon incorporating Ile into the model, the direct PM_2.5_-MetS association was no longer significant (95% CI: −0.007, 0.016). This may suggest that the statistical association between PM_2.5_ and MetS status can be entirely explained by Ile. In Table 3 and Supplementary Table S7, PM_2.5_-MA30 were positively correlated with serum Gln levels (*β* = 0.001, 95% CI: 0.0003, 0.002), whilst lower Gln levels were associated with the presence of MetS (*β* = −0.763, 95% CI: −1.339, −0.188). When Gln was included as the mediating variable, the indirect association was −0.001 and was not significant (95% CI: −0.003, 0.000). These results did not provide statistical support for a mediating role of Gln in the cross-sectional association between PM_2.5_-MA30 exposure and MetS status.

**Table 3:** Mediation effect model.

Amino acids	Association component	Estimate	Boot SE	Boot 95% CI	
				BootLL CI	BootUL CI
Gln	Direct association	0.013	0.006	0.002	0.024
	Indirect association	−0.001	0.001	−0.003	0.000
Ile	Direct association	0.005	0.006	−0.007	0.016
	Indirect association	0.007	0.002	0.003	0.010

1, PM_2.5_lag30; 2, Gln; 3, Ile; 4, MetS; Mediation effect model adjusted for general demographic characteristics: gender, age, educational attainment, marital status; lifestyle factors: smoking status, drinking status, and regular physical exercise; health behaviour factors (BMI, kidney function status, LDL-C, serum ALT, serum AST, TC); Dietary frequency survey (weekly intake frequency of fresh vegetables, fresh fruit, meat, eggs and dairy products, soy products, garlic, chilli peppers, and fried foods).

### The moderating effects of age and dietary patterns on the relationship between PM_2.5_-MA30 and MetS

To explore whether the statistical association involving Ile varied by age or dietary patterns, we conducted a moderated mediation analysis, adjusting for all confounding factors. As shown in Table 4, age did not significantly moderate the association between PM_2.5_-MA30 and Ile (interaction term: 0.000, 95%CI: −0.0001, 0.0001), nor the association between Ile and MetS (interaction term: −0.033, 95% CI: −0.113, 0.047). Furthermore, the indirect association for the “PM_2.5_ (MA30)-Ile-MetS” link was statistically significant across all age groups. However, comparisons of indirect associations between different age groups revealed no significant differences (all 95%CIs included 0). This indicates that although the data suggested an indirect statistical link across different age groups, the strength of this link did not vary significantly with age. In summary, the analysis did not find evidence that age significantly moderated the “PM_2.5_ (MA30)-Ile-MetS” indirect statistical link.

**Table 4:** Age and dietary pattern adjustment effects.

Moderator	Estimate	SE	95% Boot CI
Age and PM_2.5_-Ile	0.000	0.0001	−0.0001, 0.0001
Age and Ile-MetS	−0.033	0.041	−0.113, 0.047
Conditional direct associations			
a	0.003	0.009	−0.014, 0.019
b	0.005	0.006	−0.007, 0.016
c	0.007	0.008	−0.009, 0.023
Conditional indirect associations			
a	0.008	0.003	0.003, 0.014
b	0.007	0.003	0.003, 0.011
c	0.005	0.003	0.001, 0.011
Comparison of indirect associations between groups			
a*b	−0.001	0.002	−0.005, 0.002
a*c	−0.003	0.004	−0.010, 0.005
b*c	−0.001	0.002	−0.005, 0.002
Dietary pattern 1 and PM_2.5_-Ile	0.001	0.001	−0.0003, 0.002
Dietary pattern 1 and Ile-MetS	−0.104	0.279	−0.651, 0.443
Conditional direct associations			
a	0.008	0.008	−0.007, 0.023
b	0.004	0.006	−0.007, 0.016
c	0.001	0.008	−0.016, 0.017
Conditional indirect associations			
a	0.007	0.002	0.002, 0.011
b	0.007	0.002	0.003, 0.011
c	0.007	0.003	0.002, 0.013
Comparison of indirect associations between groups			
a*b	0.0003	0.001	−0.003, 0.003
a*c	0.001	0.003	−0.006, 0.007
b*c	0.0002	0.002	−0.003, 0.004
Dietary Pattern 2& PM_2.5_-Ile	−0.0003	0.001	−0.001, 0.001
Dietary Pattern 2&Ile-MetS	−0.091	0.349	−0.776, 0.594
Conditional direct associations			
a	0.010	0.009	−0.007, 0.027
b	0.005	0.006	−0.007, 0.016
c	−0.001	0.009	−0.018, 0.016
Conditional indirect associations			
a	0.007	0.003	0.002, 0.013
b	0.007	0.002	0.003, 0.011
c	0.006	0.003	0.001, 0.012
Comparison of indirect associations between groups			
a*b	−0.001	0.002	−0.005, 0.004
a*c	−0.002	0.004	−0.010, 0.007
b*c	−0.001	0.002	−0.005, 0.004

All model adjusted for general demographic characteristics: gender, age, educational attainment, marital status; lifestyle factors: smoking status, drinking status, and regular physical exercise; health behaviour factors (BMI, kidney function status, LDL-C, serum ALT, serum AST, TC); Dietary frequency survey (weekly intake frequency of fresh vegetables, fresh fruit, meat, eggs and dairy products, soy products, garlic, chilli peppers, and fried foods). Dietary pattern 1 represents the Hunan cuisine pattern (flavour-intensive, balanced meat-and-vegetable regimen); dietary pattern 2 denotes the fried soy products pattern. “a” indicates that the level of the moderator variable is mean-1SD; “b” indicates the mean level of the moderator variable; “c” indicates that the level of the moderator variable is mean+1SD.

A principal component analysis was conducted on the frequency of participants’ intake of fresh vegetables, fresh fruit, meat/eggs/dairy products, soy products, garlic, chillies and fried foods over the past week, resulting in the identification of two dietary patterns: the Hunan cuisine pattern (a diet characterised by heavy use of seasonings and a balanced mix of meat and vegetables) and the fried soya products pattern (e.g., fried stinky tofu, fermented bean curd and sugar-coated fried dough) (Supplementary Table S8). The results showed that the “Hunan cuisine pattern” did not significantly moderate the association between PM_2.5_-MA30 and Ile levels (interaction term: 0.001, 95%CI: −0.0003, 0.002), nor did it significantly moderate the association between Ile and MetS status (interaction term: −0.104, 95%CI: −0.651, 0.443). Similarly, the “fried soy products pattern” did not show a significant moderating link with the “PM_2.5_ (MA30)-Ile” or “Ile-MetS” associations (interaction term: −0.0003, 95%CI: −0.001, 0.001; interaction term: −0.091, 95%CI: −0.776, 0.594). Furthermore, although the indirect associations for the “PM_2.5_ (MA30)-Ile-MetS” link were significant across different dietary patterns, between-group comparisons of these indirect associations revealed no significant differences (all 95%CIs included 0). This suggests that different dietary patterns did not significantly moderate the strength of this indirect statistical link.

In summary, within this study population, we found no evidence that age or dietary patterns significantly altered the statistical mediation pattern observed for the “PM_2.5_ (MA30)-Ile-MetS” association.

## Discussion

In this exploratory cross-sectional study, we observed a weak statistical association between recent (30-day) PM_2.5_ exposure levels and current MetS status in a population of community-dwelling older adults. However, this statistical association persisted even after adjusting for various traditional metabolic pathway indicators, suggesting that the relationship between recent PM_2.5_ exposure and MetS status may be mediated by non-traditional metabolic pathways. To further explore the potential biological link underlying this cross-sectional association, we conducted a mediation analysis based on the cross-sectional study. The results revealed that serum isoleucine (Ile) levels statistically explained part of the cross-sectional association between PM_2.5_(MA30) and MetS status, thereby providing preliminary, population-based cross-sectional evidence in support of the theoretical pathway “PM_2.5_ exposure-amino acid metabolism disruption-metabolic abnormalities,” thereby narrowing a broad theoretical mechanism down to a specific, empirically testable epidemiological hypothesis and clearly pointing the way for future prospective studies.

This study investigated the association between PM_2.5_ exposure across different time windows and the presence of MetS in the older population. Notably, after adjusting for general demographic characteristics, lifestyle and behavioural factors, dietary factors, and traditional metabolic risk factors, a weak independent positive association was observed for the 30-day moving average window (PM_2.5_MA30). This weak association should be cautiously interpreted as a preliminary indication that the observed PM_2.5_-MetS link might be statistically mediated, in part, through pathways such as amino acid metabolism, rather than reflecting a broad overall association. This limited time-window specificity may reflect a variety of reasons. From a methodological perspective, a shorter exposure window may be insufficient to accumulate systemic metabolic changes detectable by current models, whilst a longer window may further dilute the already weak independent association signal due to increased cumulative measurement errors, individual adaptive responses, or complex fluctuations in exposure history. It is worth noting that the 30-day exposure window observed in this study is comparable in timescale to the subchronic exposure period (approximately 4–8 weeks) required for PM_2.5_ to induce metabolic disturbances in animal experiments (65, 66). This correspondence provides a potential, albeit yet to be verified, biological temporal reference for the weak associations identified in this study This suggests that, in a cross-sectional study, PM_2.5_ exposure most strongly associated with current metabolic status may have been concentrated in the subacute period prior to the survey. This may reflect the exacerbating effect of recent exposure on pre-existing metabolic disorders, rather than being a cause of MetS. This pattern of results may tentatively suggest that the time window for PM_2.5_ exposure to progress from causing pulmonary oxidative stress and inflammation to a pathological stage affecting the stability of systemic glucose and lipid metabolism may be 30 to 60 days. However, the impact of earlier periods of air pollution exposure on health may be obscured or attenuated due to measurement errors in cumulative exposure and individual physiological factors (67, 68). The strength of the association shows a progressive decline as the exposure timeframe increases, this may be related to reduce measurement accuracy of long-term exposure, exposure confounding effects, or the activation of partial compensatory mechanisms within the body (69, 70). The findings of this study merely provide a relevant reference for selecting exposure time assessment indicators in future related research. At the same time, the limited effect sizes observed in this study suggest that PM_2.5_ may be merely one of several environmental factors within the complex pathogenic network of MetS, with a limited independent contribution, or that its effects may be diluted by factors such as the accuracy of exposure assessment. Consequently, the findings of this study primarily serve as a reference for the selection of exposure duration assessment metrics in future research; their specific public health implications require further clarification in conjunction with more precise individual exposure assessments and longitudinal study data.

Ile, identified as a statistical mediator in our analysis, may exert its action through the known association between branched-chain amino acid metabolism disorders and insulin sensitivity. This study found a positive correlation between PM_2.5_ exposure and serum Ile concentration, whilst elevated Ile levels were associated with increased MetS risk. In recent years, elevated circulating branched-chain amino acid levels have emerged as prospective biomarkers of metabolic health (24, 27). The precise mechanism underpinning PM_2.5_-induced elevations in serum Ile requires deeper biological elucidation. Existing literature indicates that the reactive oxygen species production and systemic low-grade inflammation induced by chronic PM_2.5_ exposure may intensify proteolysis in skeletal muscle (71), leading to increased release of BCAAs, notably Ile (72). Furthermore, recent toxicological evidence demonstrates that long-term PM_2.5_ exposure directly impairs hepatic metabolic homeostasis by inducing endoplasmic reticulum (ER) stress (e.g., suppression of GRP78) and disrupting lipid clearance (73). Since the liver is the central hub for both lipid and amino acid metabolism, this PM_2.5_-induced hepatic stress may simultaneously blunt the enzymatic breakdown of Ile, exacerbating its systemic accumulation. BCAAs may also enhance immune responses by regulating cytokine secretion (74).

The underlying mechanism linking this Ile accumulation to MetS may involve excessive branched-chain amino acids and their metabolites abnormally activating the mammalian target of rapamycin complex 1 (mTORC1) signalling pathway (75). Persistent activation of this pathway disrupts normal phosphorylation of insulin receptor substrates (76), thereby impairing insulin signalling in peripheral tissues. Crucially, mTORC1 hyperactivation is intimately coupled with downstream lipogenic cascades. Recent studies highlight the HSP90β/SREBP axis as a master transcriptional pathway driving *de novo* lipogenesis and aggravating insulin resistance (77). High circulating Ile can robustly stimulate SREBP via mTORC1, driving massive hepatic lipid synthesis. Consequently, this leads to the pathological over-secretion of atherogenic lipoproteins. Indeed, comprehensive metabolomic cohort studies have established that early elevations in such lipid components-specifically very-low-density lipoproteins (VLDL) and intermediate-density lipoproteins (IDL)-are the strongest independent predictors for transitioning from a metabolically healthy state to a metabolically unhealthy phenotype (78). This study is the first to establish a link between environmental PM_2.5_ exposure, accumulated Ile, and MetS risk in the older population. It suggests that a pathway involving branched-chain amino acid metabolism may underlie the mechanism by which environmental exposure influences disease risk, with Ile emerging as a particularly crucial amino acid in this context. The association patterns revealed by our mediation analysis are consistent with our hypothesis: isoleucine, as part of the branched-chain amino acids, may represent a potential pathway between environmental exposure and metabolic dysregulation. However, due to the inherent limitations of cross-sectional studies, our findings can only indicate the potential existence of a statistical mediating pattern, rather than a causal mediating effect. Therefore, the role of Ile should be regarded as a statistically significant and biologically plausible candidate mediator. These results are primarily intended to inform specific hypotheses for future longitudinal and mechanistic studies aimed at investigating the causal relationship and biological mechanisms of the “PM_2.5_ exposure-Ile-MetS” pathway.

### Limitations

This study focuses on community-dwelling older adults, a group at high risk of MetS. The findings therefore have public health implications for the health management of this population. However, our study also has several limitations: 1) Limitations of the study design. This study employed a cross-sectional design. Although we explored potential mechanisms through mediation analyses, we were unable to confirm the temporal sequence or causal relationship between PM_2.5_ exposure, changes in amino acid levels, and the onset of metabolic syndrome. The observed associations may be confounded by unmeasured factors. Although we explored the statistical pathways between PM_2.5_, amino acids and MetS through mediation analysis, the cross-sectional study design prevents us from inferring the temporal sequence or causal relationships among the three variables. The observed associations may be influenced by unmeasured confounding factors, or there may be a possibility of reverse causality (for example, certain behaviours in patients with MetS may influence their exposure or amino acid metabolism). Consequently, the findings regarding “mediation” presented in this paper should be regarded as preliminary, hypothesis-generating evidence, and the precise causal and temporal dynamics require confirmation in prospective studies. 2) Limitations of exposure assessment. This study utilised data from fixed monitoring stations located approximately 2 km from the participants’ communities to represent individual exposure; this may result in measurement error regarding PM_2.5_ exposure concentrations. Furthermore, actual individual PM_2.5_ exposure is influenced by various factors such as the microenvironment (time spent indoors and outdoors, household ventilation, green space area, etc.) and personal protective behaviours (wearing masks); the use of fixed-site data may result in misclassification of exposure. 3) Due to objective limitations in data acquisition, we were unable to incorporate local meteorological data (such as ambient temperature and relative humidity) into the multivariate model. This may have affected the true association between PM_2.5_ and MetS. Therefore, the observed associations should be interpreted with caution, and future longitudinal studies incorporating comprehensive meteorological data are required to validate our preliminary findings. 4) Representativeness of the sample. As participants were drawn from a single community in Hengyang, China, and comprised older adults who volunteered to participate, selection bias may be present. Caution is required when generalising the study findings to other regions, younger populations, or different ethnic groups. 5) Limitations of dietary assessment. In this study, dietary habits were primarily assessed using a food frequency questionnaire (FFQ), which may be subject to recall bias and fails to precisely quantify macronutrient and micronutrient intake; this may limit the ability to assess the moderating role of diet. 6) In order to clearly assess the potential mediating role of amino acid metabolism in the association between PM_2.5_ and MetS, we must control for variables in our statistical model that may also lie downstream of PM_2.5_ and are strong determinants of MetS (such as BMI, LDL-C, TC, ALT, and AST); otherwise, these strongly associated indicators may act as confounders, severely distorting the estimation of the effect of the amino acid pathway. However, this necessary statistical adjustment also implies that the weak association between PM_2.5_ and MetS ultimately reported (OR = 1.012) reflects the residual conditional effect after excluding the influence of these indicators, rather than the overall effect of PM_2.5_ on MetS. 7) Discussion of mechanisms. Although this study identified a mediating role for isoleucine at the population level, there is a lack of in-depth molecular biological evidence to support the specific mechanisms underlying the “PM_2.5_ (MA30)-Ile” and “Ile-MetS” associations. Further validation and exploration are required using cellular experiments and animal models.

Based on the findings and limitations of this study, future research could focus on the following areas: 1) Conducting prospective cohort studies to replicate and validate the association and temporal relationship between PM_2.5_ exposure, serum Ile levels, and new cases of metabolic syndrome, thereby strengthening causal inferences. 2) Utilising more precise individual exposure assessment techniques, such as personal portable PM_2.5_ monitoring devices, to reduce exposure misclassification. 3) Expand the study population and conduct multicentre, multi-regional studies to examine the impact of geographical, climatic and pollution composition differences on the aforementioned associations. 4) Conduct intervention studies to explore whether, in environments with high PM_2.5_ exposure, nutritional supplementation or lifestyle changes can effectively reduce branched-chain amino acid levels, thereby preventing or improving the risk of MetS. 5) Utilise animal models to control PM_2.5_ exposure and dynamically monitor Ile levels and related metabolic changes in blood and tissues to elucidate the precise molecular mechanisms.

## Conclusion

This study was conducted among community-dwelling older adults in Hengyang City, Hunan Province, China. This study utilised a cross-sectional survey and controlled for a range of traditional metabolic risk factors, including BMI, TC, LDL-C, ALT and AST, in the statistical model. This analytical strategy was designed to statistically control for the influence of several known metabolic pathways, thereby attempting to isolate any residual statistical association that might be indicative of other, non-traditional pathways linking PM_2.5_ exposure to MetS status. This represents the residual statistical association between PM_2.5_ and the current status of MetS after adjustment, which may suggest that, over a 30-day exposure period, PM_2.5_ may be associated with the current status of MetS in older adults via biological pathways independent of those linked to blood lipid levels and obesity. Further mediation analysis based on cross-sectional data suggests that serum isoleucine (Ile) levels can statistically account for part of the aforementioned association between PM_2.5_ and the current status of MetS. This finding provides preliminary, exploratory, population-based cross-sectional evidence for the theoretical pathway of “PM_2.5_ exposure-amino acid metabolism disruption-metabolic abnormalities,” thereby narrowing down a broad theoretical mechanism to a specific epidemiological hypothesis that can be tested in future studies. However, as this study employed a cross-sectional observational design, it was unable to determine the temporal sequence between PM_2.5_ exposure and metabolic syndrome, as well as its potential mediating variables. Consequently, it is difficult to infer a causal relationship. Although multiple confounding factors were adjusted for in the model, unmeasured lifestyle, genetic or environmental factors may still exist. Therefore, this conclusion merely provides preliminary insight into the potential mechanisms linking PM_2.5_ exposure to metabolic health, which warrants further verification. Future large-scale prospective cohort studies are required, incorporating more precise individual exposure assessments and multi-time-point biological sample collection, to confirm the temporal relationship and causal association between PM_2.5_ exposure, dynamic changes in branched-chain amino acid metabolism, and the onset of MetS. Concurrently, animal experiments and *in vitro* studies are required to elucidate the specific molecular mechanisms by which PM_2.5_ influences Ile metabolism, thereby providing a solid scientific basis for relevant health authorities to formulate targeted intervention strategies. In summary, this cross-sectional study reports a weak association between recent exposure to PM_2.5_ and current MetS status, and suggests that isoleucine may play a statistical mediating role. These findings should be regarded solely as preliminary, hypothesis-generating evidence rather than definitive causal conclusions.

## Data Availability

The original contributions presented in the study are included in the article/Supplementary material, further inquiries can be directed to the corresponding author.

## Supplementary material

SUPPLEMENTARY FIGURE S1 Inclusion and exclusion process for study subjects.

SUPPLEMENTARY FIGURE S2 Diagram of a simple mediating effects model.

SUPPLEMENTARY FIGURE S3 Diagram of a moderated mediation model.

SUPPLEMENTARY FIGURE S4 Spearman correlations among amino acids.

SUPPLEMENTARY FIGURE S5 An exploratory conceptual framework on the effects of PM_2.5_ exposure on the risk of MetS.

## Glossary

MetS: Metabolic syndrome
PM_2.5_MA7: 7-day moving average concentration of PM_2.5_
PM_2.5_MA30: 30-day moving average concentration of PM_2.5_
PM_2.5_MA90: 90-day moving average concentration of PM_2.5_
His: Histidine
3MHis: 3-methylhistidine
PEtN: Phosphoethanolamine
Arg: Arginine
Ans: Anserine
Gln: Glutamine
EtN: Ethanolamine
Cit: Citrulline
Glu: Glutamic acid
Thr: Threonine
Hcit: Homocitrulline
bAib: *β*-aminoisobutyric acid
Abu: *α*-Aminobutyric acid
Tyr: Tyrosine
Val: Valine
Leu: Leucine
Trp: Tryptophan
BMI: Body mass index
HDL: High-density lipoprotein cholesterol
AST: Aspartate transaminase
FPG: Fasting plasma glucose
Scr: Serum creatinine
PM_2.5_: Fine particulate matter
PM_2.5_MA14: 14-day moving average concentration of PM_2.5_
PM_2.5_MA60: 60-day moving average concentration of PM_2.5_
PM_2.5_MA120: 120-day moving average concentration of PM_2.5_
Hyp: Hydroxyproline
1MHis: 1-methylhistidine
Asn: Asparagine
Tau: Taurine
Ser: Serine
Gly: Glycine
Asp: Aspartic acid
Sar: Sarcosine
bAla: β-alanine
Ala: Alanine
Aad: α-aminohexanoic acid
Pro: Proline
Cys: Cysteine
Met: Methionine
Ile: Isoleucine
Phe: Phenylalanine
Lys: Lysine
LDL: Low-density lipoprotein cholesterol
ALT: Alanine transaminase
TC: Total cholesterol
TG: Triglycerides
