# Correction: Association between PM_2.5_ exposure and metabolic syndrome in older population and the mediating effect of amino acids

**DOI:** 10.3389/fpubh.2026.2008715

**Published:** 2026-10-21

**Authors:** Xu Liu, Yunxia Zhou, Yingjie Li, Mu Wang

**Affiliations:** Epidemiology Teaching and Research Office of School of Public Health and Clinical Research Institute of Affiliated Nanhua Hospital, Hengyang Medical School, University of South China, Hengyang, Hunan, China

**Keywords:** amino acids, isoleucine, mediation analysis, metabolic syndrome, PM_2.5_

There was a mistake in Table 1 as published. In Table 1, the $\bar{x}\pm s$ value for age was erroneously written as 69.610 ± 8.980. The corrected Table 1 appears below.

**Table 1:** General characteristics of the study population (*N* = 1,506).

Characteristics	Classification	$\bar{x}\pm s$	*N* (%)
PM_2.5_lag7		16.566 ± 7.764	–
PM_2.5_lag14		16.408 ± 8.093	–
PM_2.5_lag30		20.372 ± 11.798	–
PM_2.5_lag60		25.080 ± 14.817	–
PM_2.5_lag90		28.138 ± 13.806	–
PM_2.5_lag120		33.752 ± 16.060	–
Age (years)		72.155 ± 7.113	**–**
BMI		23.407 ± 3.187	–
ALT (U/L)		21.736 ± 15.456	–
AST (U/L)		22.687 ± 10.299	–
LDL–C(mmoL/L)		3.450 ± 35.862	–
TC (mmol/L)		4.579 ± 0.960	–
Fresh vegetables		8.677 ± 4.383	
Fresh fruit		4.431 ± 2.745	
Meat, eggs and dairy		7.570 ± 4.452	
Soy products		2.759 ± 1.899	
Fried foods		0.628 ± 3.930	
Garlic		6.461 ± 4.168	
Chilli peppers		5.728 ± 4.089	
MetS	Yes	–	396 (26.295%)
	No	–	1,110 (73.705%)
Gender	Male	–	645 (42.829%)
	Female	–	861 (57.171%)
Renal function status	Normal	–	1,168 (77.556%)
	Mild decline	–	260 (17.264%)
	Significant decline	–	78 (5.179%)
Regular exercise	Yes	–	1,246 (82.736%)
	No	–	260 (17.264%)
Drinking status		–	
	Yes	–	134 (8.898%)
	No	–	1,372 (91.102%)
Smoking status	Smoking all the time	–	80 (5.312%)
	Have quit smoking	–	221 (14.675%)
	Never smoked	–	1,205 (80.013%)
Status of education	Primary school and below	–	329 (21.846%)
	Junior high school	–	726 (48.207%)
	High school/vocational high Schools	–	351 (23.307%)
	college or above	–	88 (5.843%)
	unknown	–	12 (0.797%)
Marital status	Unmarried	–	13 (0.863%)
	Married	–	1,237 (82.138%)
	Divorced/widowed	–	256 (16.999%)

SD, standard deviation; ALT, alanine aminotransferase; AST, aspartate aminotransferase; LDL-C, low-density lipoprotein cholesterol; TC, total cholesterol; PM_2.5_, fine particulate matter; PM_2.5_(lag7), PM_2.5_ lagged by 7 days; PM_2.5_(lag14), PM_2.5_ lagged by 14 days PM_2.5_(lag30), PM_2.5_ lagged by 30 days; PM_2.5_(lag60), PM_2.5_ lagged by 60 days; PM_2.5_(lag90), PM_2.5_ lagged by 90 days; PM_2.5_(lag120), PM_2.5_ lagged by 120 days; MetS, metabolic syndrome.

The original version of this article has been updated.

