# Supplementary material for: Association between PM2.5 exposure and metabolic syndrome in older population and the mediating effect of amino acids
Source: Front Public Health. 2026 Jun 18;14:1811045. doi: 10.3389/fpubh.2026.1811045 (PMC13323689; doi:10.3389/fpubh.2026.1811045)
Supplement: Supplementary file 1 [file Supplementary_File_1.DOCX]

**Supplementary table 1. Descriptive statistics of serum amino acid concentrations in study populations^1^**

| Amino acids | Mean (μg/ml) | | SD (μg/ml) |
| --- | --- | --- | --- |
| His | | 16.399 | 5.395 |
| Hyp | | 5.511 | 7.827 |
| 3MHis | | 0.493 | 0.876 |
| 1MHis | | 0.997 | 0.693 |
| PEtN | | 0.355 | 0.345 |
| Asn | | 7.812 | 2.135 |
| Arg | | 22.125 | 18.791 |
| Tau | | 26.946 | 17.435 |
| Ans | | 0.366 | 0.556 |
| Ser | | 21.517 | 12.545 |
| Gln | | 79.872 | 19.024 |
| Gly | | 28.649 | 13.891 |
| EtN | | 1.220 | 1.539 |
| Asp | | 6.451 | 3.324 |
| Cit | | 10.658 | 9.779 |
| Sar | | 0.151 | 0.068 |
| Glu | | 21.108 | 9.394 |
| bAla | | 0.370 | 0.150 |
| Thr | | 16.806 | 4.429 |
| Ala | | 46.789 | 15.078 |
| Hcit | | 0.105 | 0.103 |
| Aad | | 0.227 | 0.272 |
| bAib | | 0.343 | 0.541 |
| Pro | | 22.039 | 7.560 |
| Abu | | 3.696 | 5.929 |
| Cys | | 20.344 | 18.897 |
| Tyr | | 17.218 | 12.287 |
| Met | | 4.148 | 1.099 |
| Val | | 34.660 | 16.575 |
| Ile | | 10.034 | 2.788 |
| Leu | | 20.895 | 5.499 |
| Phe | | 19.256 | 16.010 |
| Trp | | 16.242 | 15.551 |
| Lys | | 30.803 | 8.014 |

^1^Abbreviations: Histidine, His; hydroxyproline, Hyp; 3-methylhistidine, 3MHis; 1-methylhistidine, 1MHis; phosphoethanolamine, PEtN; asparagine, Asn; arginine, Arg; taurine, Tau; anserine, Ans; serine, Ser; glutamine, Gln; glycine, Gly; ethanolamine, EtN; aspartic acid, Asp; citrulline, Cit; sarcosine, Sar; glutamic acid, Glu; β-alanine, bAla; threonine, Thr; alanine, Ala; Homocitrulline, Hcit; α-aminohexanoic acid, Aad; β-aminoisobutyric acid, bAib; proline, Pro; α-Aminobutyric acid, Abu; cysteine, Cys; tyrosine, Tyr; methionine, Met; valine, Val; isoleucine, Ile; leucine, Leu; phenylalanine, Phe; tryptophan, Trp; lysine, Lys.

**Supplementary table 2. Testing for multicollinearity among covariates**

| Variable | VIF |
| --- | --- |
| PM2.5（MA30） | 1.035 |
| age | 1.338 |
| gender | 1.643 |
| BMI | 1.089 |
| ALT | 3.814 |
| AST | 3.648 |
| LDL-C | 1.009 |
| Renal function status | 1.127 |
| Regular exercise | 1.049 |
| drinking status | 1.150 |
| smoking status | 1.423 |
| status of education | 1.168 |
| marital status | 1.141 |
| Fresh vegetables | 1.705 |
| Fresh fruit | 1.275 |
| Meat, eggs and dairy | 2.197 |
| Soy products | 1.138 |
| fried foods | 1.012 |
| garlic | 1.138 |
| chilli peppers | 2.089 |

**Supplementary table 3. The association between PM_2.5_ (MA30) and metabolic syndrome after adjusting for co-pollutants^1^**

|  | air pollution | *β* | SE | OR Value | 95%CI | *P* |
| --- | --- | --- | --- | --- | --- | --- |
| Mode 1 | PM_2.5_（MA30） | 0.012 | 0.006 | 1.012 | 1.001-1.023 | 0.033 |
|  | SO_2_（MA30） | -0.010 | 0.018 | 0.990 | 0.957-1.025 | 0.584 |
| Mode 2 | PM_2.5_（MA30） | 0.012 | 0.006 | 1.012 | 1.001-1.023 | 0.031 |
|  | NO_2_（MA30） | -0.006 | 0.019 | 0.994 | 0.959-1.032 | 0.765 |
| Mode 3 | PM_2.5_（MA30） | 0.018 | 0.006 | 1.018 | 1.005-1.030 | 0.005 |
|  | O_3_（MA30） | -0.006 | 0.003 | 0.994 | 0.988-1.000 | 0.050 |

^1^All models were uniformly adjusted for the same confounding factors, including gender, age, educational attainment, marital status, smoking status, drinking status, regular physical exercise, BMI, renal function status, LDL-C, serum ALT and AST, TC, and weekly frequency of consumption of fresh vegetables, fresh fruit, meat/eggs/dairy products, soy products, garlic, chilli peppers, and fried foods.

**Supplementary table 4 Results of elastic network regression screening for amino acids associated with PM_2.5_ exposure^2^**

| Amino acids | coefficient |
| --- | --- |
| Hyp | 0.726 |
| bAla | 7.433 |
| Arg | 2.571 |
| Gln | 0.553 |
| Glu | -7.539 |
| Cys | 1.548 |
| Tyr | 1.322 |
| Ile | 3.175 |
| Trp | 0.847 |
| Lys | 2.355 |

^2^Abbreviations: hydroxyproline, Hyp; β-alanine, bAla; arginine, Arg; glutamine, Gln; glutamic acid, Glu; cysteine, Cys; tyrosine, Tyr; isoleucine, Ile; tryptophan, Trp; lysine, Lys.

**Supplementary table 5 Elastic Network Regression Screening Results for MetS-Associated Amino Acids^3^**

| Amino acids | coefficient |
| --- | --- |
| 1MHis | -0.124 |
| Gln | -0.076 |
| Gly | -0.251 |
| Thr | -0.445 |
| Ala | 0.599 |
| Met | -0.584 |
| Val | 0.256 |
| Ile | 0.542 |
| Leu | 0.041 |

^3^Abbreviations: 1-methylhistidine, 1MHis; glutamine, Gln; glycine, Gly; threonine, Thr; alanine, Ala; methionine, Met; valine, Val; isoleucine, Ile; leucine, Leu.

**Supplementary table 6. Regression Analysis of the Isoleucine Intermediate Effect Model^4^**

| Predictor variable | Model1(Ile） | | |  | Model2(MetS） | | |
| --- | --- | --- | --- | --- | --- | --- | --- |
|  | β | t | 95% BootCI |  | β | Z | 95% BootCI |
| PM_2.5_ | 0.005 | 10.583 | 0.004, 0.006 |  | 0.005 | 0.824 | -0.007, 0.016 |
| Ile |  |  |  |  | 1.216 | 4.108 | 0.636, 1.796 |
| $R^{2}$/ McFadden | 0.167 | | |  | 0.179 | | |

^4^Effect values are standardised numerical values. All model adjusted for general demographic characteristics: gender, age, educational attainment, marital status; lifestyle factors: smoking status, drinking status, and regular physical exercise; health behaviour factors (BMI, kidney function status, LDL-C, serum ALT, serum AST, TC); Dietary frequency survey (weekly intake frequency of fresh vegetables, fresh fruit, meat, eggs and dairy products, soy products, garlic, chilli peppers, and fried foods).

**Supplementary table 7. Regression Analysis of the Glutamine Intermediate Effect Model^5^**

| Predictor variable | Model1(Gln） | | |  | Model2(MetS） | | |
| --- | --- | --- | --- | --- | --- | --- | --- |
|  | β | t | 95% BootCI |  | β | Z | 95% BootCI |
| PM_2.5_ | 0.001 | 2.486 | 0.0003, 0.002 |  | 0.013 | 2.333 | 0.002, 0.024 |
| Gln |  |  |  |  | -0.763 | -2.602 | -1.339, -0.188 |
| $R^{2}$/ McFadden | 0.038 | | |  | 0.173 | | |

^5^Effect values are standardised numerical values. All model adjusted for general demographic characteristics: gender, age, educational attainment, marital status; lifestyle factors: smoking status, drinking status, and regular physical exercise; health behaviour factors (BMI, kidney function status, LDL-C, serum ALT, serum AST, TC); Dietary frequency survey (weekly intake frequency of fresh vegetables, fresh fruit, meat, eggs and dairy products, soy products, garlic, chilli peppers, and fried foods).

**Supplementary table 8. Principal Component Analysis of Dietary Patterns: Rotated Component Matrix^6^**

| Dietary habits | Component | |
| --- | --- | --- |
|  | 1 | 2 |
| fresh vegetables | 0.753 | \ |
| fresh fruit | 0.569 | \ |
| meat, eggs and dairy products | 0.839 | \ |
| soy products | \ | 0.728 |
| garlic | 0.804 | \ |
| chilli peppers | 0.787 | \ |
| fried foods |  | 0.567 |
| Kaiser-Meyer-Olkin Measure | 0.792 | |
| Bartlett’s Test of Sphericity | *P*<0.001 | |
