# Supplementary figures and images for: Association between PM2.5 exposure and metabolic syndrome in older population and the mediating effect of amino acids
Source: Front Public Health. 2026 Jun 18;14:1811045. doi: 10.3389/fpubh.2026.1811045 (PMC13323689; doi:10.3389/fpubh.2026.1811045)

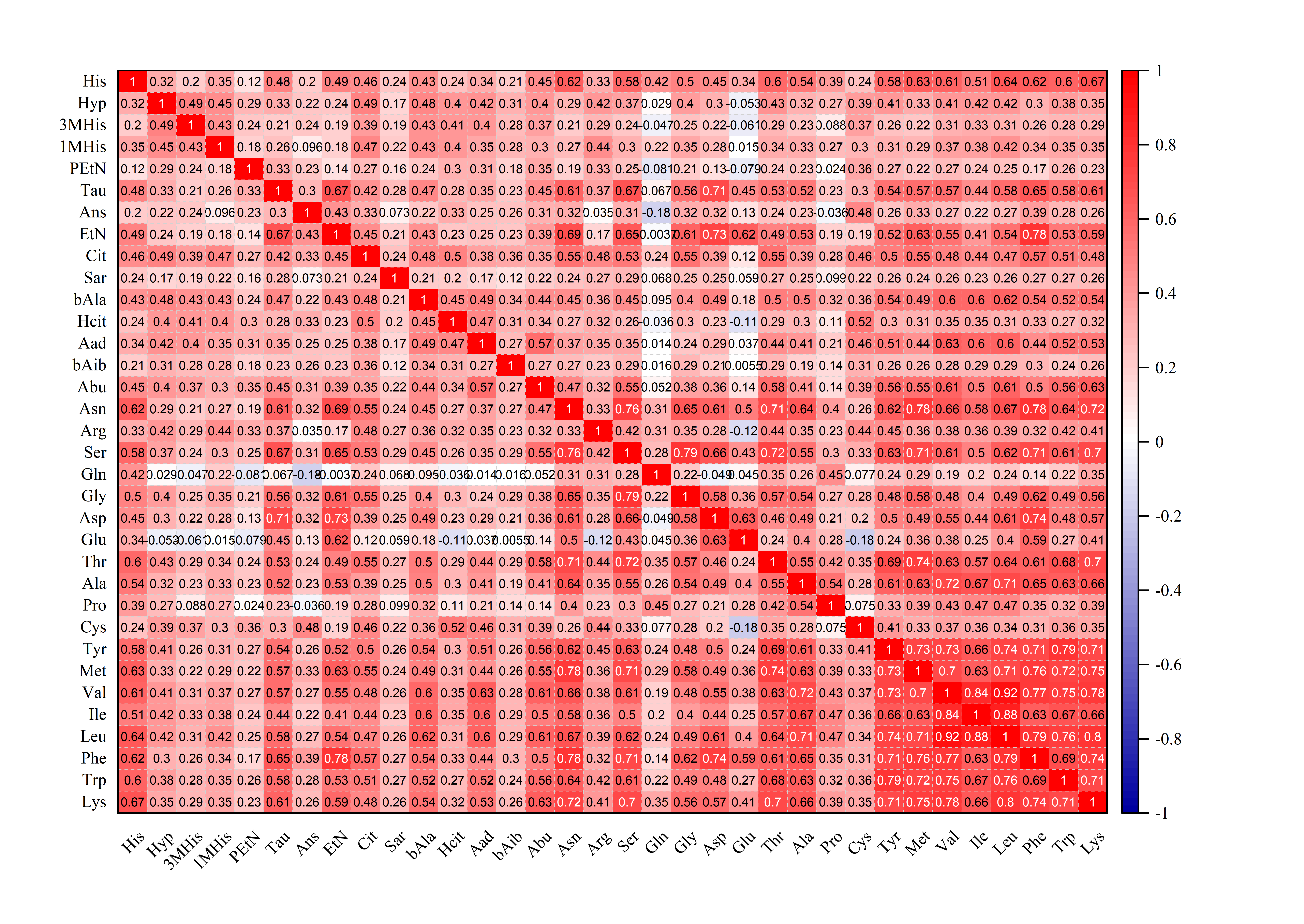

Supplement: SUPPLEMENTARY FIGURE S3 — Diagram of a moderated mediation model. [file Image_3.TIF]

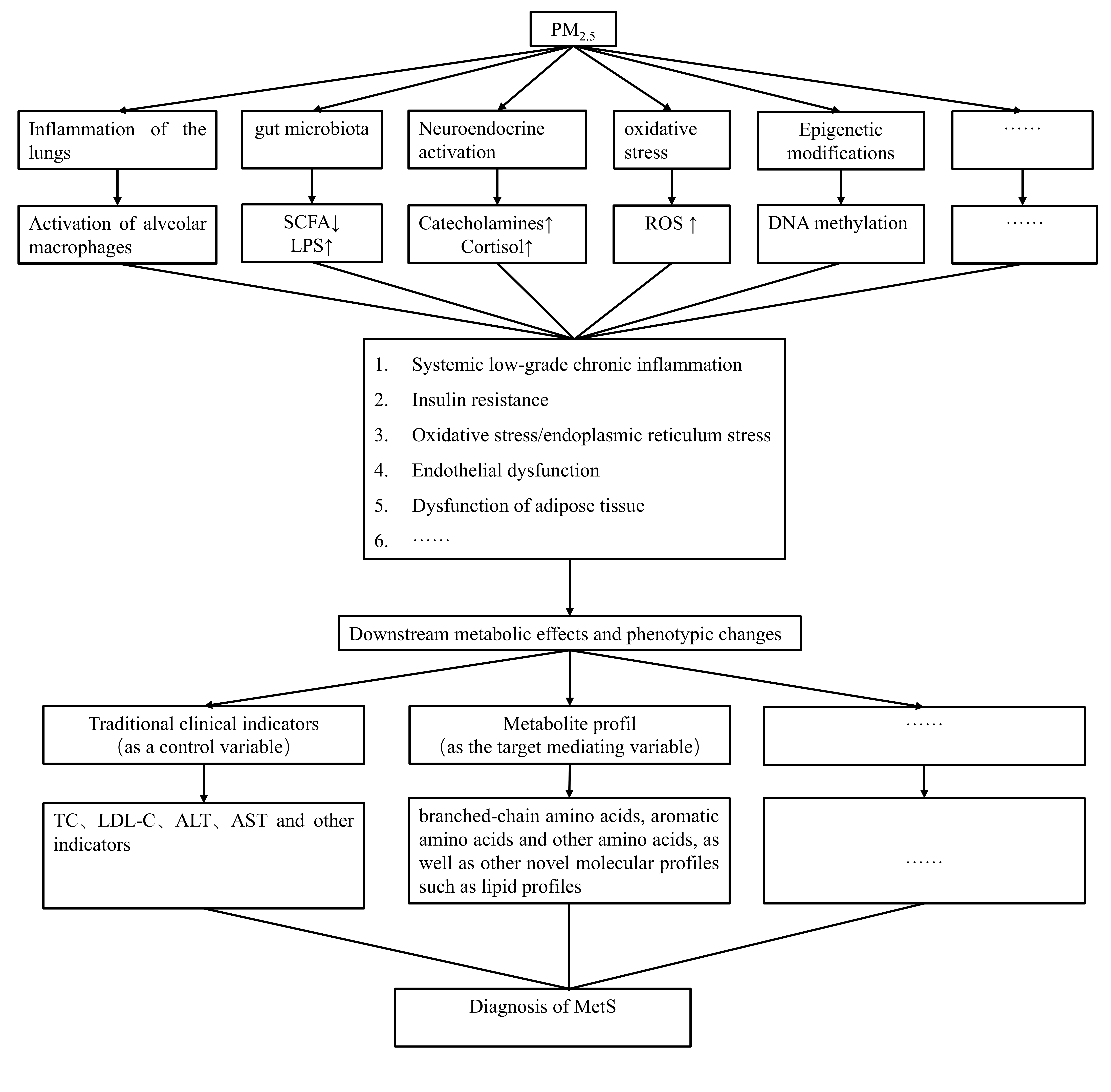

Supplement: SUPPLEMENTARY FIGURE S4 — Spearman correlations among amino acids. [file Image_4.TIF]

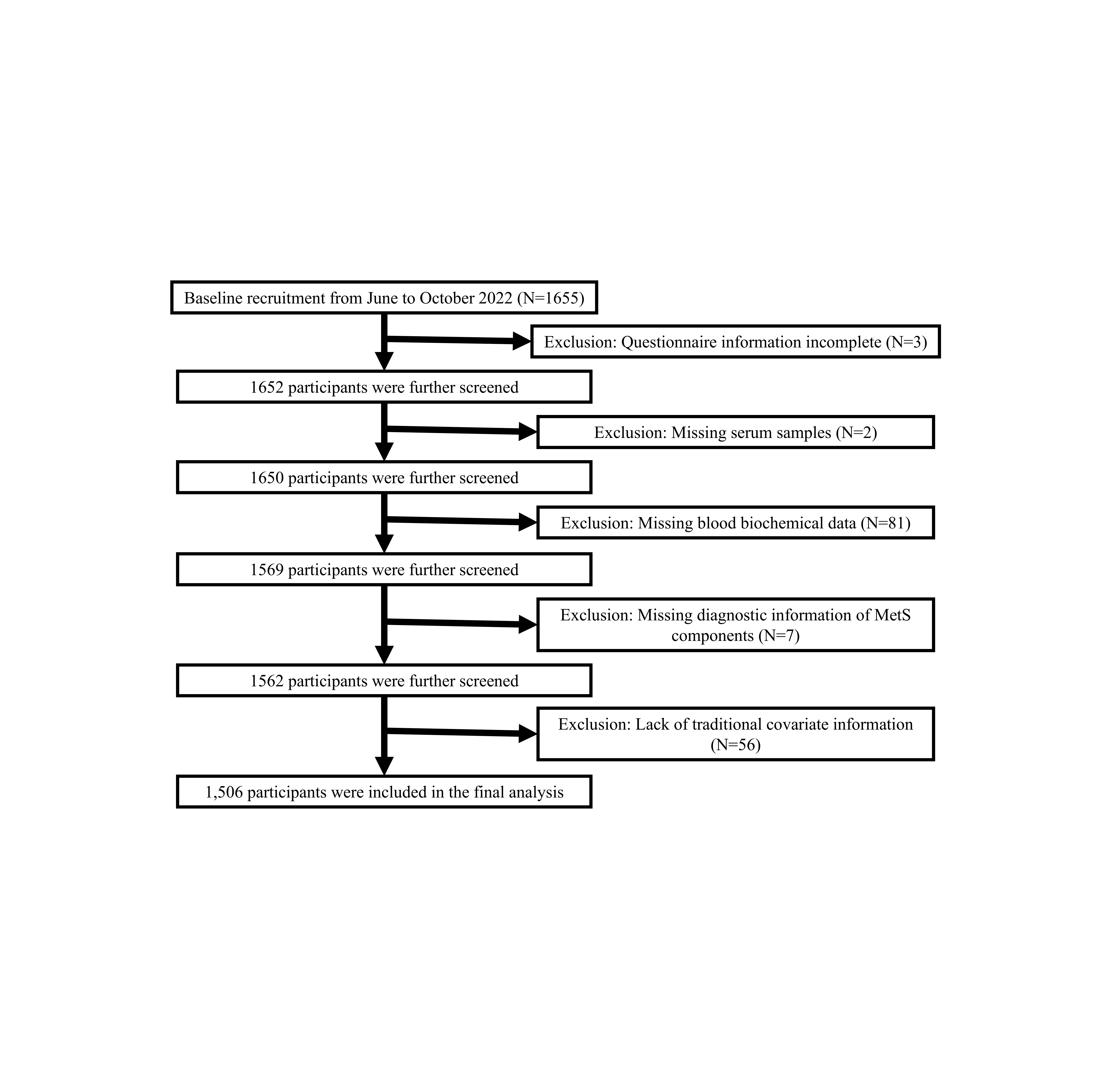

Supplement: SUPPLEMENTARY FIGURE S5 — An exploratory conceptual framework on the effects of PM2.5 exposure on the risk of MetS. [file Image_5.TIF]
